# Fungi and Archaea Control Soil N_2_O Production Potential in Chinese Grasslands Rather Than Bacteria

**DOI:** 10.3389/fmicb.2022.844663

**Published:** 2022-05-16

**Authors:** Lei Zhong, Jinwu Qing, Min Liu, Xiaoxian Cai, Gaoyuan Li, Frank yonghong Li, Guanyi Chen, Xingliang Xu, Kai Xue, Yanfen Wang

**Affiliations:** ^1^School of Environmental Science and Engineering, Tianjin University, Tianjin, China; ^2^Key Laboratory of Ecosystem Network Observation and Modeling, Institute of Geographic Sciences and Natural Resources, Chinese Academy of Sciences, Beijing, China; ^3^School of Ecology and Environment, Inner Mongolia University, Hohhot, China; ^4^Chinese Academy of Sciences (CAS) Center for Excellence in Tibetan Plateau Earth Sciences, Chinese Academy of Sciences, Beijing, China; ^5^College of Life Sciences, University of Chinese Academy of Sciences, Beijing, China

**Keywords:** nitrification, denitrification, fungi, archaea, bacteria

## Abstract

Nitrous oxide (N_2_O) is a powerful greenhouse gas and the predominant stratospheric ozone-depleting substance. Soil is a major source of N_2_O but remains largely uncertain due to the complicated processes of nitrification and denitrification performed by various groups of microbes such as bacteria, fungi, and archaea. We used incubation experiments to measure the total fungal, archaeal, and bacterial N_2_O production potential and the microbial functional genes in soils along 3,000 km Chinese grassland transect, including meadow steppe, typical steppe, desert steppe, alpine meadow, and alpine steppe. The results indicated that fungi, archaea, and bacteria contributed 25, 34, and 19% to nitrification and 46, 29, and 15% to denitrification, respectively. The AOA and AOB genes were notably correlated with the total nitrification enzyme activity (TNEA), whereas both *narG* and *nirK* genes were significantly correlated with total denitrification enzyme activity (TDEA) at *p* < 0.01. The correlations between AOA and ANEA (archaeal nitrification enzyme activity), AOB and BNEA (bacterial nitrification enzyme activity), and *narG, nirK*, and BDEA (bacterial denitrification enzyme activity) showed higher coefficients than those between the functional genes and TNEA/TDEA. The structural equation modeling (SEM) results showed that fungi are dominant in N_2_O production processes, followed by archaea in the northern Chinese grasslands. Our findings indicate that the microbial functional genes are powerful predictors of the N_2_O production potential, after distinguishing bacterial, fungal, and archaeal processes. The key variables of N_2_O production and the nitrogen (N) cycle depend on the dominant microbial functional groups in the N-cycle in soils.

## Introduction

Net primary productivity is usually restricted by nitrogen bioavailability in the terrestrial ecosystem (LeBauer and Treseder, 2008). Nonetheless, a fraction of available N can be lost *via* the N_2_O flux from the soil, further aggravating the limitation of N. The emission of N_2_O from the soil is mainly caused by the activity of nitrifiers and denitrifiers and contributes to 57% of the global N_2_O emissions (Tian et al., 2019). As a powerful greenhouse gas, the warming potential of N_2_O is 300-fold stronger than that of carbon dioxide (Ravishankara et al., 2009). Consequently, several studies have been conducted to explore the emission of N_2_O from terrestrial soils to determine its production and budget on a regional scale (Ravishankara et al., 2009; Attard et al., 2011; Cantarel et al., 2012; Zhong et al., 2014; Tian et al., 2019). However, global N_2_O budgets remain largely uncertain due to the complicated microbial processes involving N_2_O flux from soils (Skiba and Smith, 2000).

The nitrification and denitrification processes have been gradually identified (Arnold, 1954); for example, ammonia is converted to nitrite and then to nitrate by nitrifiers during nitrification. Subsequently, ${\text{NO}}_{3}^{-}$ can be reduced to NO${\text{NO}}_{2}^{-}$ and then to NO and is finally transformed into N_2_O and N_2_ by denitrifiers during denitrification (Offre et al., 2013). Initially, microbial nitrification and denitrification were hypothesized to be mainly controlled by bacteria through functional genes (Table 1) (Stein, 2020). Most of these studies have been conducted in croplands or grasslands, which were managed or intermediate environments because the environments are more suitable for bacterial growth (Francis et al., 2007; Klotz and Stein, 2008). Meanwhile, more and more researches have been conducted to determine the relationship between microbial functional gene abundance and N_2_O emission to accurately predict soil N_2_O fluxes (Saleh-Lakha et al., 2009; Li et al., 2021). However, consistent relationships are seldom observed between them, suggesting that the gene abundance was not facilitating the prediction of N_2_O fluxes from the soil (He et al., 2010; Attard et al., 2011; Zhong et al., 2014; Kou et al., 2019).

**Table 1:** Enzymes and functional genes involved in microbial production and consumption of N_2_O.

**Progress**	**Organism**	**Enzyme**	**General reaction**	**Gene name(s)**
**Nitrification**	AOB, AOA, comammox	Ammonia monooxygenase	NH_3_ → ${\text{NO}}_{2}^{-}$	*amo*
	AOB, comammox, anammox	Hydroxylamine dehydrogenase	NH_2_OH → NO	*hao*
	Comammox, NOB	Nitrite oxidoreductase	${\text{NO}}_{2}^{-}$ → ${\text{NO}}_{3}^{-}$	*nxr*
**Denitrification**	Denitrifiers, denitrifying methanotrophs, ANME-2d	Nitrate reductase (dissimilatory)	${\text{NO}}_{3}^{-}$ → ${\text{NO}}_{2}^{-}$	*nar*
	Denitrifiers, denitrifying methanotrophs, NC10, AOB, AOA, comammox, NOB, anammox	Nitrite reductase (dissimilatory)	${\text{NO}}_{2}^{-}$ → NO	*nirS, nirK*
	Denitrifiers, denitrifying methanotrophs, (some) AOB	Nitric oxide reductase	NO → N_2_O	*nor*
	Denitrifiers, nondenitrifying N_2_O reducers	Nitrous oxide reductase	N_2_O → N_2_	*nosZ*

Recent research has reported that fungi and archaea also participate in the production of N_2_O, following similar pathways to those of bacterial nitrification/denitrification. However, the process of fungal nitrification is still unclear, and fungal denitrification does not involve the reduction of nitrous oxide due to the absence of the *nos* gene (Wankel et al., 2017). Since the ammonia-oxidizing archaea were first observed in the oceans, archaeal nitrification has gained widespread attention and proved crucial in many habitats (Treusch et al., 2005; Leininger et al., 2006; Francis et al., 2007). Furthermore, Zhu et al. (2015) has also demonstrated that fungi also play an important role in nitrification and dominate the heterotrophic nitrification of acidic soils. Furthermore, the contribution of fungal denitrification to the N_2_O production potential ranged from 17.0 to 89.1% in dryland or soil with high organic matter content (Zhong et al., 2018). This indicates that fungi and archaea could also play important roles in nitrification and denitrification due to their various environmental adaptabilities. However, few studies have quantified the contribution of bacteria, fungi, and archaea to N_2_O production and their driving factors (Xu et al., 2017; Kaurin et al., 2018). Thus, the knowledge of the driving mechanisms of N_2_O flux is limited, and the functional genes involved in soil N_2_O fluxes are not well known. This might explain why previous studies using only the bacterial functional genes were unable to reach total N_2_O production in the soil (Zhong et al., 2018).

Grassland accounts for about 20% of the global terrestrial land (DAHV CISNR, 1996). Grassland, which is the third largest ecosystem, occupies 41.70% of the land in China, with the presence of diverse grasslands and soil types across the north and northwest parts of China under various climates (DAHV CISNR, 1996; Wang and Fang, 2009). This provides a unique platform to examine N_2_O production and clarify the roles of various microbial functional groups on a large scale (Liao and Jia, 1996). Previous studies demonstrated that soil moisture was the major factor in the N-cycle in Chinese grasslands due to the arid and semiarid climates, but the dominant microbes in soil N_2_O production remain unknown (Zhong et al., 2014). To understand the role of bacteria, fungi, and archaea in the soil N-cycle, we quantified their contributions to N_2_O production *via* the nitrification and denitrification processes and determined the relationships between archaeal, fungal, or bacterial functional genes and N_2_O production potential in Chinese grassland soils over a large space. Based on previous studies, we hypothesize the following: (1) fungi and archaea play a major role in soil N_2_O production since climatic and edaphic conditions are more suitable for them in the grasslands of Northern China (Zhong et al., 2014, 2018); (2) the differentiation of the bacterial, fungal, and archaeal N_2_O production processes can increase the predictive power of microbial functional genes in soil N_2_O production. Finally, we have also clarified the driving factors responsible for the production of N_2_O in Chinese grasslands and have demonstrated that integrating the roles of bacteria, fungi, and archaea will remarkably improve the evaluation of the soil N_2_O budget.

## Materials and Methods

### Chinese Grassland Transects and Soil Sampling

Soil samples were collected from four stations in the Inner Mongolia grassland and four in the Tibet grassland (Supplementary Table S1), spanning nearly 20° in latitude (31°26′N−50°21′N), 30° in longitude (89°02′E−119°07′E), and 4,000 m in altitude range (618–4,700 m above sea level). The mean annual air temperature (MAT) on the grasslands varied from −2.6 to 2.1°C and −2.1 to 1.1°C in Inner Mongolia and Tibet grasslands, respectively. The mean annual precipitation (MAP) varied from 223 to 385 mm and 310 to 630 mm in the Inner Mongolia and Tibet grasslands, respectively (Supplementary Table S1).

The main grassland types in Inner Mongolia were meadow, typical, and desert steppe, which were from northeast to southwest. The typical steppe, also known as true steppe or dry steppe, occupies the largest and most contiguous extent of all the steppe ecosystem types in China and holds the central position in the ecological sequence of Chinese steppe grassland ecosystems (Li et al., 2020). The major grassland types in the Tibet Plateau were alpine meadow steppe, alpine steppe, and alpine desert steppe. The details of dominant plant species and soil types were the same as mentioned by Kou et al., 2019.

The soil samples were collected in triplicate in August 2017. Each time, five samples were mixed in equal proportions by three soil cores (10 cm in diameter) at a depth of 0–10 cm taken from each plot located along a diagonal line. All soil samples were sieved, homogenized, and then divided into two fractions: one was air-dried for chemical analyses, and the other was preserved at −20°C for 2 weeks for enzyme and gene abundance analysis.

### DNA Extraction and Measurement

To determine the soil levels of ${\text{NO}}_{3}^{-}$-N and ${\text{NH}}_{4}^{+}$-N, the soil sample was extracted with KCl solution (2 mol/L), and then an Automated Ion Analyzer (Quickchem FIA Star 5010, LACHAT) was utilized. Gravimetric soil moisture (SM) was determined by the percentage of moisture content in the soil after oven-drying at 105°C for 24 h. The soil level analyses of total nitrogen (TN) and total carbon (TC) were carried out by the Kjeldahl method and the H_2_SO_4_-K_2_Cr_2_O_7_ oxidation, respectively (Nelson et al., 2015).

We extracted soil DNA using the MoBio–DNA Kit according to the manufacturers' instructions. The DNA samples were kept at −20°C before the determination. The population sizes of ammonia monooxygenase, *amoA*-AOA, *amoA*-AOB, bacterial *nirK, nirS*, and *nosZ* clade I and II genes and of fungal *nirK* (*FnirK*) were measured using Q-PCR in triplicates. The primers are shown in Supplementary Table S2, and the method has been described in the study by Zhong et al. (2017, 2018).

### The Nitrification Enzyme Activities

To determine the N_2_O production potential from the total and the individual potentials of bacteria, fungi, and archaea, we determined the total (TNEA), bacterial (BNEA), fungal (FNEA), and archaeal (ANEA) nitrification enzyme activities using an improved method of Dassonville et al. (2011). In a 250 ml bottle, we added 80 ml of solution of (NH_4_)_2_SO_4_ (50 μg N- g^−1^ dry soil) to offer the ${\text{NH}}_{4}^{+}$-N as the raw material for the nitrification process, then an equivalent amount of 10 g of fresh soil to dry soil was added. Each soil sample was divided into four treatment groups with three replications [all inhibitors' concentrations were determined by inhibitor additivity ratios (IAR) evaluation]: (1) bactericide (streptomycin sulfate, C_42_H_84_N_14_O_36_S_3_) at 3.0 mg g^−1^; (2) fungicide (cycloheximide, C_15_H_23_NO_4_) at 1.5 mg g^−1^ (Castaldi and Smith, 1998; Laughlin and Stevens, 2002); (3) sterilized group (0.3 MPa and 121°C for 30 min) (Heil et al., 2015); and (4) no-inhibitor control. Then they were kept on a shaker at 28°C (180 rpm) for incubation, and a 10 ml of sample (soil slurry) was taken and filtered at 0, 24, and 48 h. An automated discrete analyzer (Smartchem 200, LACHAT) was used to analyze the ${\text{NO}}_{3}^{-}$ + ${\text{NO}}_{2}^{-}$ density. Here, we define the NEA as the nitrification enzymatic activity rate, and k is equal to the slope of the time-dependent linear rate of the ${\text{NO}}_{2}^{-}+$${\text{NO}}_{3}^{-}$ generation. Thus, TNEA (total NEA) = k (no-inhibitor control); BNEA (bacteria NEA) = TNEA – k (bactericide group); FNEA (fungi NEA) = TNEA – k (fungicide group); ANEA (archaea NEA) = TNEA – BDEA – FDEA – k (sterilized group).

### The Denitrification Enzyme Activities

To determine the N_2_O production potential from the total and the individual potentials of the bacteria, fungi, and archaea, we determined the total (TDEA), bacterial (BDEA), fungal (FDEA), and archaeal (ADEA) denitrification enzyme activities using the method proposed by Patra et al. (2006) and Marusenko et al. (2013). In a 250 ml bottle, we added 80 ml of solution of KNO_3_ (50 μg N- g^−1^ dry soil), glutamic acid (0.5 mg C- g^−1^ dry soil), and glucose (0.5 mg C- g^−1^ dry soil) as raw materials for the denitrification process, then an equivalent amount of 10 g of fresh soil to dry soil was added. Each soil sample was divided into four treatment groups with three replications (all inhibitors' concentrations were determined by IAR evaluation): (1) bactericide (streptomycin sulfate, C_42_H_84_N_14_O_36_S_3_) at 3.0 mg g^−1^; (2) fungicide (cycloheximide, C_15_H_23_NO_4_) at 1.5 mg g^−1^; (3) sterilized group (0.3 MPa and 121°C for 30 min); and (4) no-inhibitor control. Also, the air at the bottle headspaces was replaced by the N_2_ gas and C_2_H_2_ (10% v/v) to suppress the N_2_O-to-N_2_ reduction and maintain an anaerobic denitrification process. Then they were kept in a shaker at 28°C (180 rpm) for incubation, and a 10 ml gas sample was taken at 0, 24, and 48 h during the period and then used to analyze the N_2_O concentration *via* gas chromatography. Here, we define the DEA as the denitrification enzymatic activity rate, and k is equal to the slope of the time-dependent linear rate of the N_2_O generation. Thus, TDEA (total DEA) = k (no-inhibitor control); BDEA (bacteria DEA) = TDEA – k (bactericide group); FDEA (fungi DEA) = TDEA – k (fungicide group); ADEA (archaea DEA) = TDEA – BDEA – FDEA – k (sterilized group).

### Inhibitor Additivity Ratios Evaluation

The concentrations of inhibitors mentioned above were determined using IAR evaluation (Bailey et al., 2003; Zhong et al., 2022). Therefore, it is sure that the concentrations of inhibitors are sufficient to achieve the best inhibition and target the microbe without affecting other types of microorganisms. The incubation experimental conditions of the IAR evaluation are the same as those of the enzyme activity experiment. The IAR is estimated using the equation: IAR = [(A – B) + (A – C)] / (A – D). A is the no-inhibitor group, B is the bactericide group, C is the fungicide group, and D is the bactericide and fungicide group. Their values are equal to the slope of the time-dependent linear rate of the ${\text{NO}}_{3}^{-}$ + ${\text{NO}}_{2}^{-}$ density or N_2_O generation. The results of IAR are shown in Supplementary Table S3.

### Verification of the Archaeal Nitrification and Denitrification Enzyme Activity

The archaeal nitrification and denitrification enzyme activities were estimated by calculating the difference between all treatments due to the lack of specific inhibitors for archaea. Zhao et al. (2020) reported simvastatin as a specific inhibitor of archaea; hence, we compared the accuracy of determining the archaeal nitrification and denitrification enzyme activities by the two methods.

We experimented with the parts of the “The nitrification enzyme activities” and “The denitrification enzyme activities” sections and added another treatment (5), wherein simvastatin (C_25_H_38_O_5_, an archaea code) at 12.5 mg g^−1^ in solution was used to inhibit the nitrification and denitrification activities of the soil archaea. The nitrification and denitrification enzyme activities of the soil archaea were estimated by the difference between the rates of denitrification enzyme activity under treatments (4), (3), (2), and (1) as ANEA1 and ADEA1 and between treatments (4) and (5) as ANEA2 and ADEA2. The results are presented in Supplementary Figure S1 to document the accuracy of our method.

### Statistical Analyses

The random forest (RF) analysis was used to explore the most important predictors influencing the N_2_O production potentials from nitrification and denitrification, using the program “random forest” in the statistical package R (Liaw and Wiener, 2002). The details were described in the study by Archer, 2021 and Evans and Murphy, 2019.

For a better understanding of the chemical, physical, and biological traits of soil, as well as the N_2_O generation potentials, the structural equation modeling (SEM) was used considering all variables (MAP, MAT, soil pH, TC, TN, ${\text{NH}}_{4}^{+}$-N, ${\text{NO}}_{3}^{-}$-N, TNEA, FNEA, ANEA, BNEA, TDEA, FDEA, ADEA, and BDEA). For a better model fit, we represented ${\text{NH}}_{4}^{+}$, ${\text{NO}}_{3}^{-}$, TC, and TN by the soil factors (SC) through the principal component analysis (PCA) aided by SPSS 18 to reduce the model variable number. Given the small sample number for the variable number per modeling (*n* = 32), the estimates were likely conservative and fit (Shipley et al., 2004; Kang and Shipley, 2009). The gene abundance was examined for statistical significance *via* one-way ANOVA; significant differences among 8 locations were further examined at a 0.05 level through Duncan's multirange test. The gene abundance was also examined for statistical significance using the Mann–Whitney U test, with significant differences between Tibet and Inner Mongolia (*p* < 0.05). The SEM model was employed in IBM^®^ SPSS^®^ AmosTM 20. The chi-squared (χ^2^) test was applied to determine the difference between actual observed data and predicted by the model, and a *p*-value > 0.05 indicated no significant difference between the predicted and actual observed data. The coefficients were estimated by standardized coefficients then examined by analyzing correlation matrices and were considered significant when *p* < 0.05 (Petersen et al., 2012).

## Results

### The Abundance of the N-Cycle Genes Across the Grassland Transect

The abundance of the functional microbial groups (bacteria: AOB, *narG, nirK, nirS, nosZ*, and *nosZ* clade II genes; archaea: AOA gene; and fungi: *nirK* gene) related to the N-cycle was measured (Table 2). However, the fungal *nirK* gene was not detected (Supplementary Table S2; Table 2). Across the grassland transect, there is a significant difference between Tibet and Inner Mongolia on AOA, AOB, *narG, nirK*, and *nirS* (*p* < 0.05, Table 2). The AOB and *narG* gene abundances ranged between 0 to 2.6 × 10^8^ and 7.9 × 10^3^ to 1.8 × 10^7^ copies g soil^−1^, respectively. The *nirK* and *nirS* genes copies ranged from 1.9 × 10^3^ copies g soil^−1^ at the Sonid Zuoq site to 1.8 × 10^7^ copies g soil^−1^ at the Hezuo site and from 1.8 × 10^3^ copies g soil^−1^ at the Sonid Zuoq site to 3.6 × 10^6^ copies g soil^−1^ at the Hulunbuir site, respectively. The abundances of the *nosZ* and *nosZ* clade II genes ranged from 4.7 × 10^6^ to 2.4 × 10^7^ and 0 to 6.8 × 10^5^ copies g soil^−1^, but the AOA gene abundance ranged from 1.6 × 10^4^ to 9.6 × 10^8^ copies g soil^−1^ across all the sites. Among 8 sites, Sonid Zuoq site is the significantly lowest (*p* < 0.05) on all gene abundance; Hezuo site is the significantly highest (*p* < 0.05) on AOA, AOB, and *narG*; Haibei site is the significantly highest (*p* < 0.05) on *nirK*; Hulunbuir site is the significantly highest (*p*< 0.05) on *nirS*; Xilin Hot site is the significantly highest (*p* < 0.05) on *nosZ*; and Haibei, Hezuo, and Nagqu are significantly higher than Sonid Zuoq on *nosZ* clade II (*p* < 0.05). The fungal *nirK* gene was not determined in any of the sites across the grassland transect.

**Table 2:** Copy numbers of soil microbial functional nitrification and denitrification genes in grasslands.

	**Gene copies g^**−1**^ soil**	AOA^*^	AOB^*^	narG^*^	nirK^*^	***nirS***	***nosZ***	***nosZ* clade II**
**Tibet**	Baingoin	4.2E8 ± 4.1E7**c**	1.3E6 ± 7.9E5**a**	5.1E6 ± 4.7E5**cd**	3.0E6 ± 5.6E5**cd**	3.0E5 ± 4.2E4**b**	3.2E4 ± 2.1E3**c**	6.3E6 ± 5.3E6**ab**
	Nagqu	6.6E8 ± 2.6 E7**d**	5.9E7 ± 2.2E7**b**	4.5E6 ± 6.0E5**cd**	5.0E6 ± 1.2E6**de**	3.6E5 ± 1.6E4**b**	1.9E5 ± 3.8E4**d**	1.6E7 ± 4.5E6**b**
	Hezuo	9.0E8 ± 7.6 E7**e**	2.6E8 ± 4.8E7**d**	1.8E7 ± 2.5E6**e**	8.1E6 ± 1.4E6**e**	1.6E6 ± 9.7E4**c**	4.3E5 ± 2.2E4**f**	1.8E7 ± 1.1E6**b**
	Haibei	9.6E8 ± 3.2 E7**e**	1.5E8 ± 1.2E7**c**	1.4E7 ± 2.5E6**e**	1.3E7 ± 1.8E6**f**	1.3E6 ± 2.4E5**c**	5.1E5 ± 3.9E4**g**	2.4E7 ± 1.3E7**b**
**Inner magnolia**	Duolun	1.6E4 ± 8.0 E3**a**	3.9E7 ± 1.2E7**b**	1.1E6 ± 9.7E5**b**	5.3E5 ± 1.1E5**b**	1.6E6 ± 5.4E5**c**	1.7E4 ± 4.0E3**b**	4.7E6 ± 1.9E6**ab**
	Sonid Zuoq	3.2E5 ± 7.4 E4**a**	0.0 ± 0.0**a**	7.9E3 ± 2.6E2**a**	1.9E3 ± 6.0E2**a**	1.8E3 ± 1.8E2**a**	0.0 ± 0.0**a**	5.1E3 ± 2.2E2**a**
	Xilin Hot	1.9E8 ± 1.8 E7**b**	7.0E7 ± 2.9E7**b**	6.8E6 ± 1.4E5**d**	2.2E6 ± 1.2E6**bc**	2.3E5 ± 5.2E4**b**	6.8E5 ± 3.0E4**h**	7.6E6 ± 3.0E6**ab**
	Hulunbuir	2.2E8 ± 4.7 E6**b**	1.6E6 ± 1.0E6**a**	3.1E6 ± 2.0E5**bc**	1.3E6 ± 2.1E5**bc**	3.6E6 ± 4.2E5**d**	3.3E5 ± 2.8E4**e**	5.7E6 ± 3.3E6**ab**

The values represent means ± 1 SEM (n = 4) and followed by a different letter are significantly different between different sites (p < 0.05). With ^*^ mark means that there is a significant difference between Tibet and Inner Mongolia via the Mann–Whitney U test (p < 0.05).

### Nitrification and Denitrification Enzyme Activities Across the Grassland Transect

**Figure 1:**
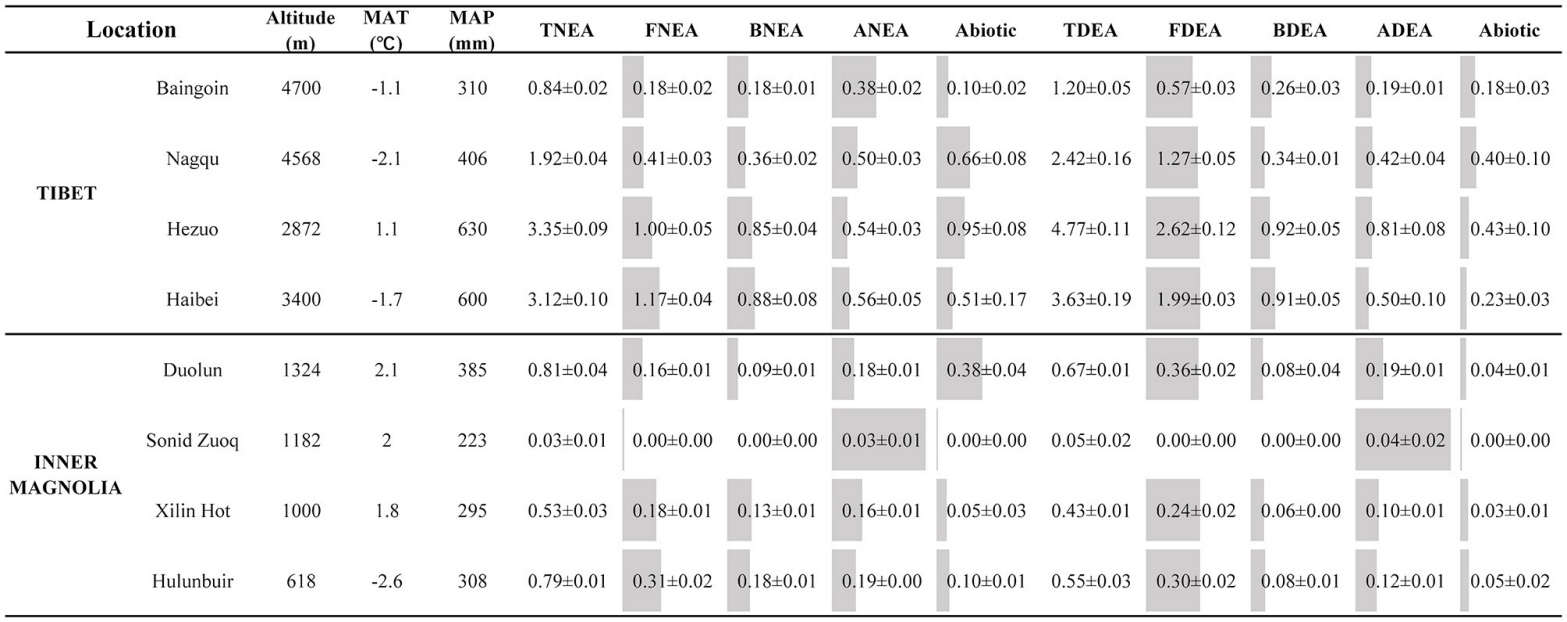
The activities of nitrification and denitrification enzymes from fungi (FNEA and FDEA), bacteria (BNEA and BDEA), and archaea (ANEA and ADEA) and total nitrification and denitrification enzymes activities (TNEA and TDEA) at different sampling sites in grassland soils. The values represent means ± 1 SEM (*n* = 4). The length of the gray bar means the contribution percentage of fungi, bacteria, archaea, and abiotic on the total nitrification or denitrification enzymes activities (0% equal to 0 cm, 100% equal to cell width).

Across the Chinese grassland transect, the TNEA and TDEA in the soils varied from 0.03 to 3.34 and 0.05 to 4.77 μg N g^−1^ h^−1^, respectively. The BNEA, FNEA, and ANEA varied from 0 to 0.88, 0 to 1.17, and 0.03 to 0.56 μg N g^−1^ h^−1^, respectively. In comparison, the BDEA, FDEA, and ADEA varied from 0 to 0.92, 0 to 2.62, and 0.04 to 0.81 μg N g^−1^ h^−1^, respectively. In the Tibetan grasslands, the NEA and DEA from bacteria, fungi, and archaea were much higher than in the Inner Mongolia grasslands, except for FNEA and BNEA in Baingoin (Figure 1).

The verification of the archaeal nitrification and denitrification enzyme activity showed no significant difference (*p* > 0.05) between these two methods, which agreed with our design and the results of the ANEA and ADEA methods.

### Correlations Between the Soil Factors and Gene Abundance, and Between the Gene Abundance and Enzyme Activities

**Figure 2:**
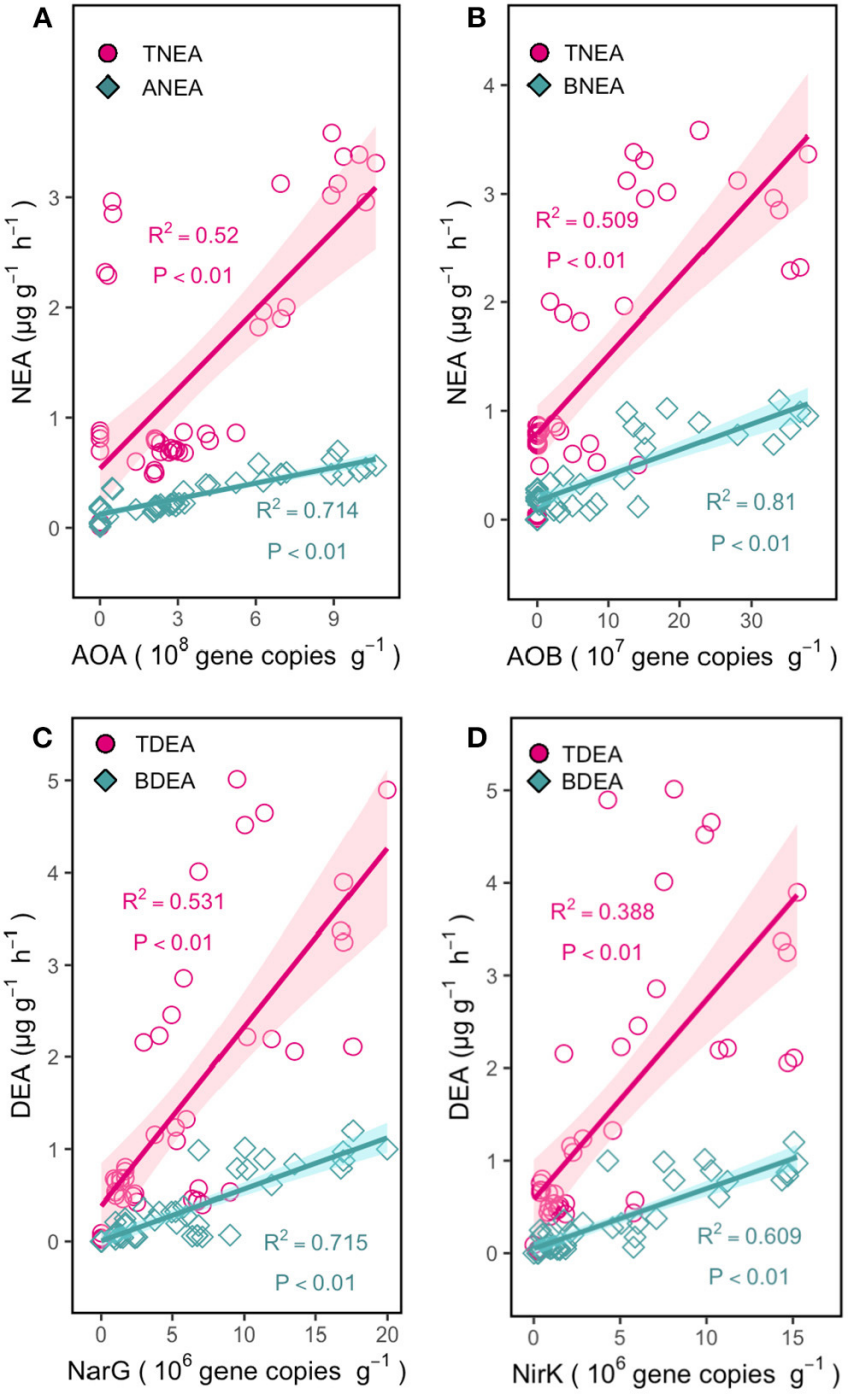
Relationships between the abundance of AOA and TNEA or ANEA **(A)**; AOB and TNEA or BNEA **(B)**; *narG* and TDEA or BDEA **(C)**; *nirK* and TDEA or BDEA **(D)** in grassland soils. The *R*^2^ values are the coefficients of determination.

**Figure 3:**
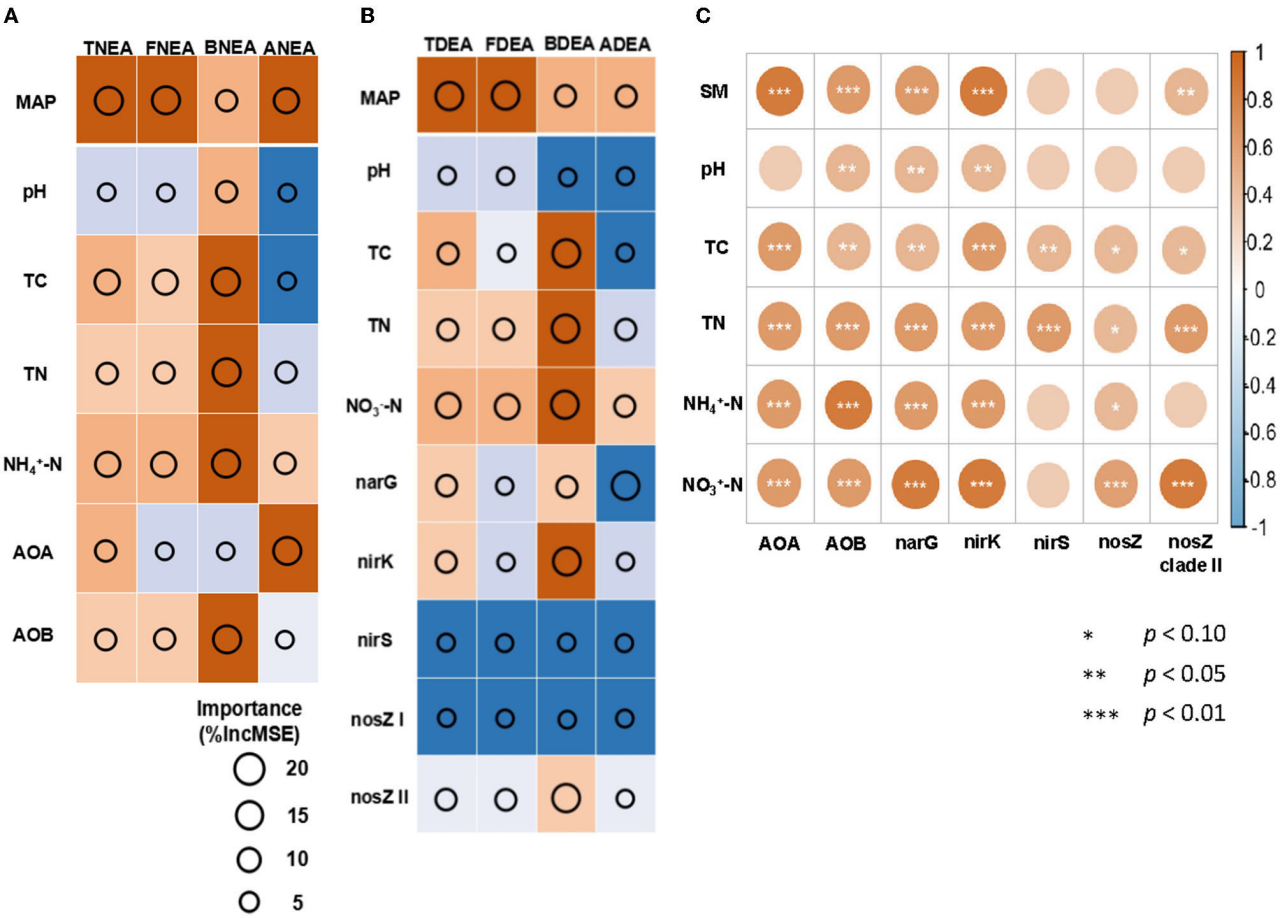
Random forest (RF) models to identify the significant environmental factors and the abundance of nitrification genes controlling TNEA, FNEA, BENA, and ANEA **(A)**; the abundance of denitrification genes controlling TDEA, FDEA, BDEA, and ADEA **(B)**. The relationships between the physical and chemical properties of the soil and gene abundance **(C)**. The importance is calculated as the percentage (%) increase in the mean square error in RF models (%IncMSE). For **(A,B)**, the size of the circles indicates the importance of the environmental factors or the gene abundance for TNEA and TDEA. The text colored in orange represents adjusted *p* < 0.05; in blue represents adjusted *p* > 0.05, with the darker color indicating the lower or higher *p*-value, respectively. For **(C)**, the color shade indicated the coefficients of determination, * indicates *p* < 0.10, ** indicates *p* < 0.05, and *** indicates *p* < 0.01.

The results revealed that the gene abundance was strongly associated with enzyme activities at the regional level, which is among 8 sites' regions (Figure 2). The abundance of AOA, AOB, *narG*, and *nirK* genes significantly correlated with all factors, except for the pH and AOA. The abundance of *nirS* significantly correlated with TC and TN. The *nosZ* gene was significantly correlated with TC, TN, ${\text{NH}}_{4}^{+}$-N, and ${\text{NO}}_{3}^{-}$-N, whereas that of *nosZ* clade II was significantly correlated with SM, TC, TN, and ${\text{NO}}_{3}^{-}$-N (Figure 3C).

Significant linear correlations were observed between the AOB genes, BNEA (*R*^2^ = 0.81, *p* < 0.01) and TNEA (*R*^2^ = 0.51, *p* < 0.01); between the AOA genes, ANEA (*R*^2^ = 0.71, *p* < 0.01) and TNEA (*R*^2^ = 0.52, *p* < 0.01); between the *narG* genes, BDEA (*R*^2^ = 0.72, *p* < 0.01) and TNEA (*R*^2^ = 0.53, *p* < 0.01); and between the *nirK* genes, BDEA (*R*^2^ = 0.61, *P* < 0.01) and TNEA (*R*^2^ = 0.39, *p* < 0.01). The predictive power of function genes was noticeable base on the significant linear correlations especially between microbial function genes with corresponding strain enzyme activities, e.g., AOA with ANEA. The *nirS, nosZ* +*nosZ* clade II gene abundances were not significantly correlated with BDEA or TDEA (Figure 2).

### Factors Controlling the Nitrification and Denitrification Process

**Figure 4:**
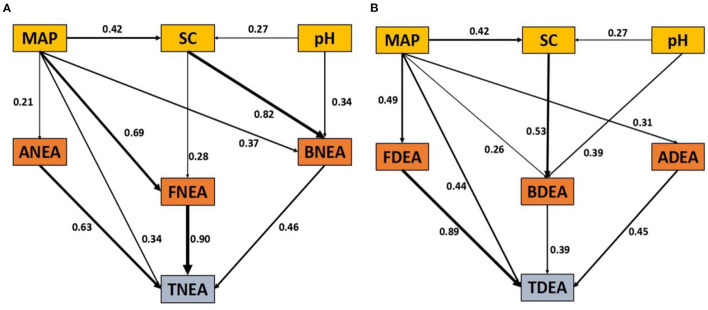
Path plot of the final model, which depicts the pattern observations in both TNEA **(A)** and TDEA **(B)**. The coefficients related with arrows represent the coefficients for multiple linear regressions. SC, soil condition; MAP, mean annual precipitation; pH, soil pH. The number of the pathway is the coefficient with significant levels denoted (*p* < 0.05).

The RF results showed that MAP was the foremost factor influencing the TNEA, FNEA, ANEA, TDEA, and FDEA. The TC, TN, and ${\text{NH}}_{4}^{+}$-N were also important influencers of BNEA, while the TC, TN, and ${\text{NO}}_{3}^{-}$-N influenced BDEA. The AOA, AOB, and *nirK* genes were the foremost factor for ANEA, BNEA, and BDEA, respectively (Figures 3A, 4B).

The soil factors (SC) were the data of TN, TC, ${\text{NH}}_{4}^{+}$-N, and ${\text{NO}}_{3}^{-}$-N by principal component analysis (Supplementary Figure S2). The first principal components explained 68.9% of the total variance, suggesting that they could sufficiently describe the change in these data as soil factors. The primary model is presented in Supplementary Figure S3. The bacterial nitrification and denitrification could be assessed indirectly by quantifying the functional genes such as AOB, *narG*, and *nirK*. However, this approach could not assess the fungal or archaeal nitrification and denitrification because there were no fungal or archaeal functional genes related to nitrification and denitrification. As a result, to equally evaluate the relative importance of bacteria, fungi, and archaea to TNEA and TDEA, we did not include functional genes in the structural equation modeling.

The SEM results demonstrated fitting of the conceptual models for both TNEA and TDEA (Figure 4A, TNEA: χ^2^ = 4.138, d.f. = 7, *p* = 0.598; Figure 4B, TDEA: χ^2^ = 5.365, d.f. = 7, *p* = 0.721) to the observation data in the conventional system. For TNEA, FNEA was the foremost factor, followed by ANEA and BNEA. FNEA was explained by MAP and SC; BNEA was explained by SC, MAP, and soil pH. Similarly, for TDEA, FDEA was the most important controlling factor, followed by ADEA and BDEA. FDEA was explained by MAP, while BDEA was explained by SC. ANEA and ADEA had no relationship with other factors.

## Discussion

### The Nitrification and Denitrification Gene Abundance

It was shown that the abundances of nitrification and denitrification genes revealed significant spatial heterogeneity in the Chinese grassland (Table 2). The abundances of the genes were higher in the Tibetan grassland as compared to the Inner Mongolian grassland, with the highest being at the Hezuo and Haibei sites and the lowest at the Sonid Zuoq site. This was in line with a previous study that also reported that the denitrification genes were the highest in the Tibetan grassland in China (Kou et al., 2019). This suggested that spatial heterogeneity may be a major driver of the changes in genes related to the nitrogen cycle. In our study, the relationship between soil moisture and AOA gene was the highest compared to that with other soil factors, whereas the bacterial nitrification and denitrification genes were more related to soil nutrients such as ${\text{NH}}_{4}^{+}$-N, ${\text{NO}}_{3}^{-}$-N, TC, or TN content, compared to soil moisture and pH (Figure 3C). All these relationships proved that the soil properties were crucial in driving the variation in the genes involved in the nitrogen cycle, but the driving factors were different for different functional genes because the responses to the changes in environmental factors may be different (Che et al., 2018; Zhong et al., 2018).

### The Contribution of Bacteria, Fungi, and Archaea to Nitrous Oxide Production Potential

Previous studies have demonstrated that fungi are powerful nitrifiers and denitrifiers in arid, semiarid, or acidic soils (Laughlin and Stevens, 2002; Marusenko et al., 2013; Zhu et al., 2015; Zhong et al., 2018), with some evidence demonstrating nitrification and denitrification by archaea (Jung et al., 2011; Li et al., 2020). However, the researchers only compared the relative importance of the bacterial and fungal N_2_O production potential or between AOA and AOB genes. The contribution of bacteria, fungi, and archaea to N_2_O production is still unclear. Here, we used soils collected along the Chinese grassland transect and incubated them to quantify their contributions. We observed that the fungi, archaea, and bacteria contributed 25, 34, and 19% to the nitrification potential and 46, 29, and 15% to the denitrification potential, respectively (Figure 1). It was confirmed that the first hypothesis is true: fungi and archaea play a major role in soil N_2_O production and further suggested that fungi play a major role in N_2_O production, followed by archaea and bacteria (Figure 4). However, fungal *nirK* and archaea denitrification genes were not found, but fungi and archaea are the important contributors to the denitrification progress, which proved that the existing primers we used (Supplementary Table S2) are not suitable in Chinese north grasslands soil; those primers are only successfully proved in agricultural soil (Duan et al., 2018; Lourenço et al., 2022; Zhong et al., 2022). It is partly in agreement with those studies that showed that the AOA was more important in nitrification than AOB in temperate grasslands (Che et al., 2018), and fungi dominated the nitrous oxide production processes in Tibet grasslands (Zhong et al., 2018). However, some researchers have found that AOA produce less N_2_O than bacteria (Giguere et al., 2017; Waggoner et al., 2021), which is in contrast with our result. We believe that the different soil conditions can explain it; AOB has higher activities than AOA under high inorganic nutrients environment or artificial pasture (Zhong et al., 2014).

Regarding the Chinese north grasslands, minor contributions from bacterial nitrifiers and denitrifiers are ascribed to the low contents of inorganic nutrients or arid environment conditions in the temperate grasslands (Zhong et al., 2014), as well as the cold climate or high organic matter content in the alpine grasslands (Zhong et al., 2018). This is because bacteria generally have high gene abundance and activities in soils with high content of inorganic nutrients or relatively high moisture (Di et al., 2009; Yang et al., 2017). The average archaeal nitrification rates were higher than fungal nitrification because FNEA and BNEA were closer to 0% and dominated by archaea due to the extreme drought at Sonid Zuoq (Supplementary Table S1). If the Sonid Zuoq site is excluded, the average FNEA would be higher than the archaeal nitrification across the Chinese grassland transect. Therefore, fungi and archaea are more important than bacteria for N_2_O production process in both temperate and alpine grasslands.

### The Prediction Ability of the Functional Gene Abundance to the Potential of Nitrous Oxide Emissions

Compared to the previous studies that used only bacterial functional genes, this study demonstrates that the gene abundance is a powerful predictor in the N_2_O production process after integrating the bacterial and fungal genes. The archaeal processes of nitrification and denitrification (Figure 2), i.e., the correlations between the AOA and ANEA, AOB and BNEA, and *narG, nirK*, and BDEA were much higher than those between AOA, AOB, and TNEA or between *narG, nirK*, and TDEA (Figure 2). The RF model also confirms these results and shows the same trend (Figure 3). It is in line with previous research, which reported that the abundances of bacterial nitrifiers and denitrifiers are weak indicators for predicting the rates of total nitrification and denitrification (Attard et al., 2011; Zhong et al., 2014; Kou et al., 2019). The microbial functional groups and soil N transformation rates and/or N_2_O emissions lack significant relationships, which is ascribed to using only the bacterial functional genes (Attard et al., 2011; Zhong et al., 2014; Kou et al., 2019). However, the contribution of bacteria to the N_2_O potential is often much lower than those of fungi and archaea, as the latter have different adaptation strategies to environmental changes compared to bacteria, i.e., inorganic nutrients can significantly increase the abundance and activity of the AOB gene but have no effects on the AOA gene in an agroecosystem (Xiang et al., 2017). Besides, warming can increase the bacteria but decrease the fungal N_2_O production potential in alpine grasslands (Zhong et al., 2018). All these studies show no or low correlation between the microbial functional genes and the total nitrification and denitrification rates. However, after we distinguished the bacterial, fungal, and archaeal N_2_O production processes, the results of correlations analysis found a significant relationship between function genes with enzyme activities. Therefore, we confirm that the functional genes are powerful indexes to the potential of nitrous oxide prediction after distinguishing the bacterial, fungal, and archaeal N_2_O production processes.

### The Driving Factors Dominant the Nitrous Oxide Production Processes

To clarify the driving factors of bacterial, fungal, and archaeal N_2_O production, the soil nitrous oxide production pathways were investigated by SEM analysis. It indicated that fungi represent a major source of N_2_O production, followed by archaea. Several previous studies suggest that MAP or soil moisture play an important role in driving nitrification and denitrification in regions across the Chinese grassland transect, but they considered only the climate or soil properties (Wang et al., 2006; Zhong et al., 2014). Our results provide a different explanation: the MAP or soil moisture drives the N_2_O production process, where fungi and archaea are the dominant microbes in the N_2_O production process because they have also been reported to be the factors in the MAP in Chinese grassland.

The RF and SEM analysis confirmed that the MAP was the most important factor for fungal and archaeal nitrification and denitrification (Figures 3, 4), which was the major contributor to the N_2_O production potential. This indicates that MAP is a major driving factor in the N_2_O production in Chinese grassland. On the other hand, our study also demonstrated that mainly bacterial nitrification and denitrification were explained by different factors compared to the processes of archaea and fungi. The BNEA and BDEA were largely controlled by SC instead of MAP at the regional scale (Figures 3, 4). This finding was different from that observed in most studies on arid or semiarid grasslands, which reported that the bacterial functional genes, communities, or activities were mainly controlled by the MAP or soil moisture, as the grassland ecosystem was considered to be limited by water (Zhong et al., 2014, 2017). This might be explained by the higher sensitivity of bacterial nitrification and denitrification to changes in nutrient composition compared to soil moisture, even in semi-arid or alpine environmental conditions (Xiang et al., 2017). Therefore, our results highlight that it is necessary to describe the dominant microorganisms of the N_2_O production process to know its driving mechanism in the ecosystem, which is fungi in our research.

**Figure 5:**
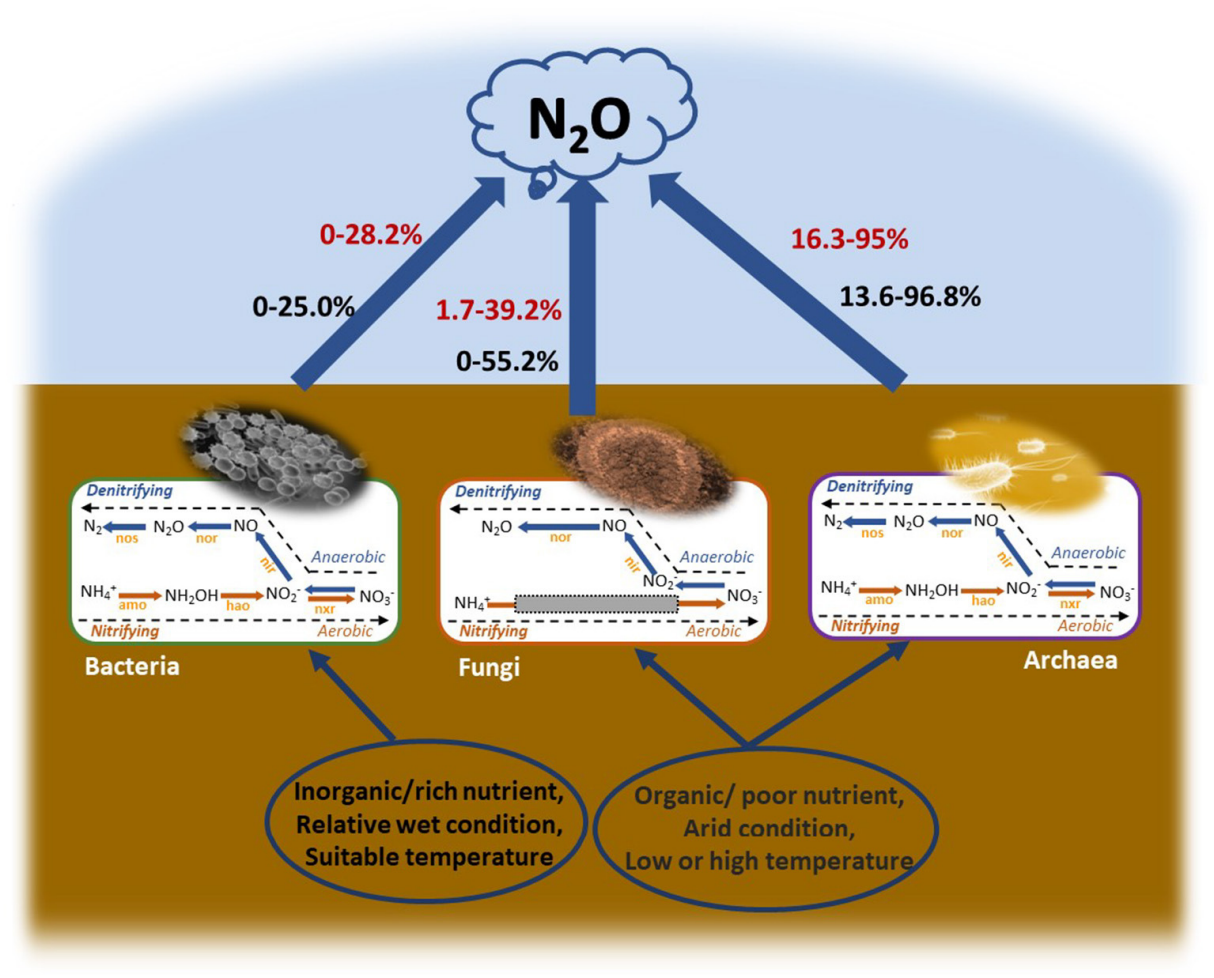
Contribution and mechanisms of bacteria, fungi, and archaea to the N_2_O production potential. The red color of the numbers represents the contribution of BNEA, FNEA, or ANEA to TNEA. The black color of the numbers represents the contribution of BDEA, FDEA, or ADEA to TDEA.

Based on the above results, if the role of fungi and archaea in N_2_O production is not accounted for, the evaluations of the budget of N_2_O from the soil will be highly uncertain. Several classical models like DAYCENT or DNDC simulated the emission of N_2_O with nitrification and denitrification modules (Ri et al., 2003; Del Grosso et al., 2005). Wu et al. (2015) used the SPACSYS model further to simulate the autotrophic/heterotrophic nitrification and denitrification and used its responses to environmental factors or nutrient availability to simulate of the N_2_O flux. All these ecosystem-modeling procedures in the soil make a unique pool; therefore, it ignored the microbial type and generally assumed that the variations in the biogeochemical process can predict system behavior based on a simple hypothesis, which simply takes all microbes as one pool and regardless of how the identity and abundance of microbial communities' changes. In our view, this “black box” may be valid only if the N-cycle process was dominated and made up largely by bacteria; otherwise, it might be a major reason behind the uncertainty of these models (Allison and Martiny, 2008). The modeling of N_2_O emission should further distinguish the bacterial, fungal, and archaeal nitrification and denitrification modules to improve the accuracy of the models (Figure 5). If so, the abundance of the microbial functional genes related to N_2_O production can significantly improve the accuracy of N_2_O prediction and can also be an important module of the models. On the contrary, while our study provides a novel insight into the models of N_2_O emission, it has several limitations. First, we only demonstrated that the microbial functional genes were powerful indicators for predicting N_2_O production potential after distinguishing the bacterial, fungal, and archaeal nitrification and denitrification. Improving the method to distinguish the N_2_O flux of bacteria, fungi, and archaea is important for improving the model. Secondly, irrespective of the metagenomic sequencing, geochip, or Q-PCR, the results were mainly based on the bacterial microbial functional genes. In contrast, the fungal, and particularly the archaeal functional genes, were studied less due to the lack of the primers for fungal *nirK* genes suitable for grassland soil and the lack of clarity regarding fungal nitrification (Vogel et al., 2009; Gao et al., 2020), and also lack of clarity regarding archaea denitrification, which only a few cultured archaea are capable of denitrification (Torregrosa-Crespo et al., 2016). Therefore, it is important to reveal the fungal nitrification process and design more primers for the fungal and archaeal functional genes related to the N_2_O production process and then promote the microbial module to improve the accuracy of the models further.

## Conclusion

We demonstrate that fungi and archaea play dominant roles in the N_2_O production process in the grasslands of North China. It is suggested that the microbial functional genes are powerful indicators for predicting N_2_O production potential after distinguishing the bacterial, fungal, and archaeal N_2_O production processes. Besides, the key controlling variable on N_2_O production and N-cycle depends on the dominant microorganisms of the N-cycle in soils. Therefore, accurate predictions for N_2_O production and contribution to the development of the ecosystem N-cycle models will benefit from distinguishing the bacterial, fungal, and archaeal N-cycle process.

## Data Availability Statement

The raw data supporting the conclusions of this article will be made available by the authors, without undue reservation.

## Author Contributions

LZ: conceptualization, methodology, and writing—original draft. JQ: investigation and writing—review and editing. ML: visualization. XC and GL: writing—review and editing. FL: data curation. GY: resources. XX: formal analysis. KX and YW: methodology, validation, and supervision. All authors contributed to the article and approved the submitted version.

## Funding

This research was funded by the Tianjin Science and Technology Committee (Grant No. 19JCQNJC13900) and the National Natural Science Foundation of China (No. 41601245).

## Conflict of Interest

The authors declare that the research was conducted in the absence of any commercial or financial relationships that could be construed as a potential conflict of interest. The reviewer KD declared a shared affiliation with the author ML, XX, and YW at the time of the review.

## Publisher's Note

All claims expressed in this article are solely those of the authors and do not necessarily represent those of their affiliated organizations, or those of the publisher, the editors and the reviewers. Any product that may be evaluated in this article, or claim that may be made by its manufacturer, is not guaranteed or endorsed by the publisher.
