# Supplementary material for: Fungi and Archaea Control Soil N2O Production Potential in Chinese Grasslands Rather Than Bacteria
Source: Front Microbiol. 2022 May 16;13:844663. doi: 10.3389/fmicb.2022.844663 (PMC9149426; doi:10.3389/fmicb.2022.844663)
Supplement: Supplementary file 1 [file Data_Sheet_1.docx]

Supplementary Material

**Table S1** Sampling site location and soil physico-chemical properties.(MAT: mean annual temperature; MAP: mean annual precipitation; SM: soil moisture; TC: total carbon; TN: total nitrogen)

| Location | | Grassland type | Longitude | Latitude | Altitude (m) | MAT (℃) | MAP (mm) | SM (%) | pH | TC (mg kg^-1^) | TN (mg kg^-1^) |
| --- | --- | --- | --- | --- | --- | --- | --- | --- | --- | --- | --- |
| Tibet | Baingoin | Alpine grassland | 31°26′ | 90°2′ | 4700 | -1.1 | 310 | 11.89±0.61 | 6.78±0.09 | 1.68±0.12 | 0.18±0.01 |
|  | Nagqu | Alpine grassland | 31°36′ | 91°29′ | 4568 | -2.1 | 406 | 17.38±0.98 | 6.23±0.05 | 3.14±0.15 | 0.26±0.01 |
|  | Hezuo | Alpine grassland | 34°58′ | 102°53′ | 2872 | 1.1 | 630 | 25.90±0.83 | 7.84±0.05 | 5.88±0.30 | 0.42±0.03 |
|  | Haibei | Alpine grassland | 37°29' | 101°12' | 3400 | -1.7 | 600 | 28.70±0.70 | 7.86±0.02 | 6.34±0.38 | 0.50±0.02 |
| Inner Mongolia | Duolun | Transition zone between cropping area and nomadic area | 42°02′ | 116°17′ | 1324 | 2.1 | 385 | 5.23±0.27 | 6.75±0.06 | 2.03±0.11 | 0.21±0.01 |
|  | Sonid Zuoq | Temperate desert grassland | 42°53′ | 114°04′ | 1182 | 2.0 | 223 | 0.94±0.03 | 7.08±0.06 | 0.83±0.02 | 0.08±0.01 |
|  | Xilin Hot | Temperate typical grassland | 44°11′ | 116°27′ | 1000 | 1.8 | 295 | 11.36±0.72 | 7.89±0.05 | 3.16±0.05 | 0.24±0.01 |
|  | Hulunbuir | Temperate meadow grassland | 49°21′ | 120°07′ | 618 | -2.6 | 308 | 13.70±0.42 | 6.74±0.07 | 5.51±0.08 | 0.46±0.01 |

Table S2 Enzymes encoded by functional genes measured in this study, and the thermal conditions and primer sequences used in qPCR.

| **Functional gene** | **Enzyme** | **Annealing time and temperature** | **Elongation time and temperature** | **Primer** | **Primer sequence** |
| --- | --- | --- | --- | --- | --- |
| **Bacterial amoA^1^** | Ammonia monooxygenase | 56°C,30s | 72°C,30s | amoA1F | GGG GTT TCT ACT GGT GGT |
|  |  |  |  | amoA2R | CCC CTC KGS AAA GCC TTC TTC |
| **Archaeal amoA^2^** | Ammonia monooxygenase | 55°C,30s | 72°C,30s | CrenamoA23F | STA ATG GTC TGG CTT AGA CG |
|  |  |  |  | CrenamoA616R | GCG GCC ATC CAT CTG TAT GT |
| **narG^3^** | Nitrate reductase | 60°C,30s | 72°C,20s | narGG-F | TAY GTS GGG CAG GAR AAA CTG |
|  |  |  |  | narGG-R | CGT AGA AGA AGC TGG TGC TGT T |
| **nirK^4^** | Nitrate reductase | 60°C,30s | 72°C,20s | nirK876 | ATY GGC GGV CAY GGC GA |
|  |  |  |  | nirK1040 | GCC TCG ATC AGR TTR TGG TT |
| **nirS^5^** | Nitrate reductase | 60°C,30s | 72°C,20s | nirS4QF | GTS AAC GYS AAG GAR ACS GG |
|  |  |  |  | nirSR3CD | GAS TTC GGR TGS GTC TTS AYG AA |
| **nosZ^5^** | Nitrous oxide reductase | 60°C,30s | 72°C,20s | nosZ2F, | CGC RAC GGC AAS AAG GTS MSS GT |
|  |  |  |  | nosZ2R | CAK RTG CAK SGC RTG GCA GAA |
| **nosZ clade II^6^** | Nitrous oxide reductase | 60°C,30s | 72°C,20s | nosZ-II-F | CTI GGI CCI YTK CAY AC |
|  |  |  |  | nosZ-II-R | GCI GAR CAR AAI TCB GTR C |
| **Fungal nirK1^7^** | Nitrate reductase | 52°C,30s | 70°C, 1 min | fnirK2F | GTY CAY ATY GCY AAC GGS ATG TAC GG |
|  |  |  |  | fnirK2F | GCR TGR TCN ACM AGN GTR CGT CCC |
| **Fungal nirK2^8^** | Nitrate reductase | 52°C,30s | 70°C, 1 min | nirKfF | TAC GGG CTC ATG tay gtn sar cc |
|  |  |  |  | nirKfR, | AGG AAT CCC ACA scn ccy ttn tc |
| **Fungal nirK3^9^** | Nitrate reductase | 52°C,30s | 72°C, 1 min | FnirK_F3 | GCA RAG CGA GTT YTA CCA YG |
|  |  |  |  | FnirK_R2 | TVC CGA TDA YRT GGA AYG ARC |

Table S3 The percentage of reduction in gas (N_2_O or CO_2_) fluxes caused by use of both streptomycin and cycloheximide (Both), and the inhibitor additivity ratios (IAR) of soils from Inner Mongolia and Tibet grassland at three sampling times.

|  | Time (hours) | Both (%) | | | IAR | | |
| --- | --- | --- | --- | --- | --- | --- | --- |
|  |  | 0 | 24 | 48 | 0 | 24 | 48 |
| Inner Mongolia grassland soil | N_2_O | 64.2 | 68.7 | 60.8 | 1.02 | 0.99 | 0.98 |
|  | CO_2_ | 64.0 | 63.9 | 49.2 | 1.04 | 1.01 | 1.00 |
| Tibet grassland soil | N_2_O | 69.2 | 70.3 | 61.0 | 1.02 | 1.07 | 0.97 |
|  | CO_2_ | 57.9 | 55.8 | 63.0 | 1.04 | 1.10 | 0.99 |

Fig. S1 The nitrification and denitrification enzyme activities of soil archaea was estimated by the difference between rates of denitrification enzyme activity under treatment (Ⅳ), (Ⅲ), (Ⅱ) and (Ⅰ) as ANEA_1_ and ADEA_1_; and (Ⅳ), (Ⅴ) as ANEA_2_ and ADEA_2_. T test showed the ANEA1 and ANEA2 (P=0.23), ADEA1 and ANEA2 (P=0.49) all were no significant differences.


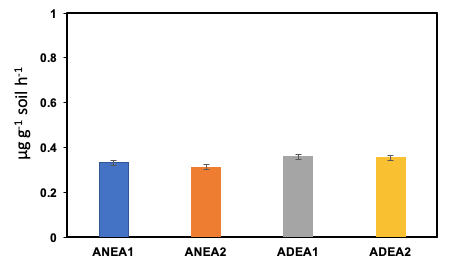


**P > 0.05**

Figure S2. Principal Component Analysis (PCA) plot showing the multivariate variation among 8 locations in terms of environmental variables. Colored symbols correspond to in this study.
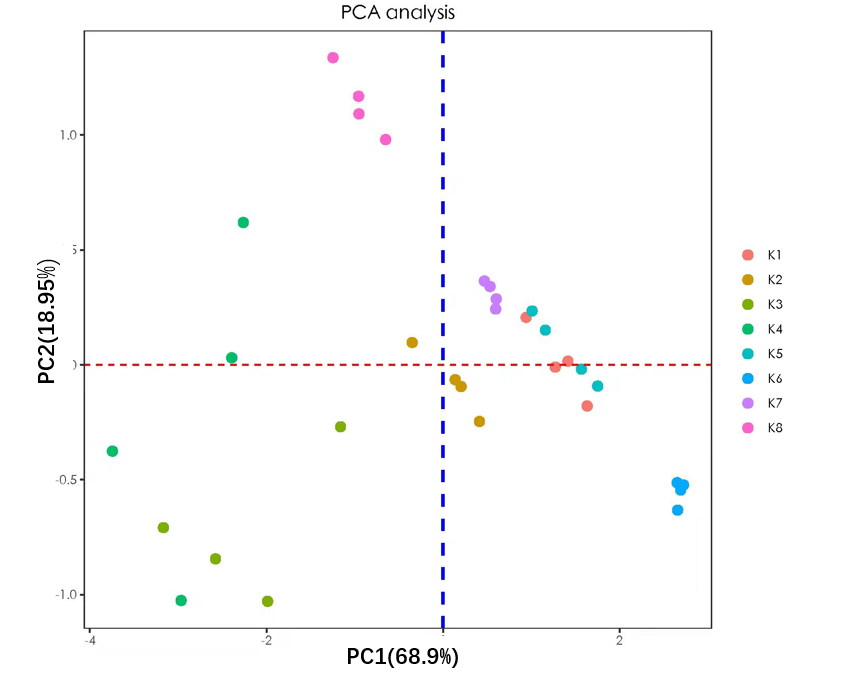


Fig. S3 A priori models for the structural equation modeling analysis of TNEA and TDEA. Arrows indicate flows of causality based on knowledge. SC– soil condition; SM – soil moisture; Temp-the average temperature of sampling site in August, 2017 .


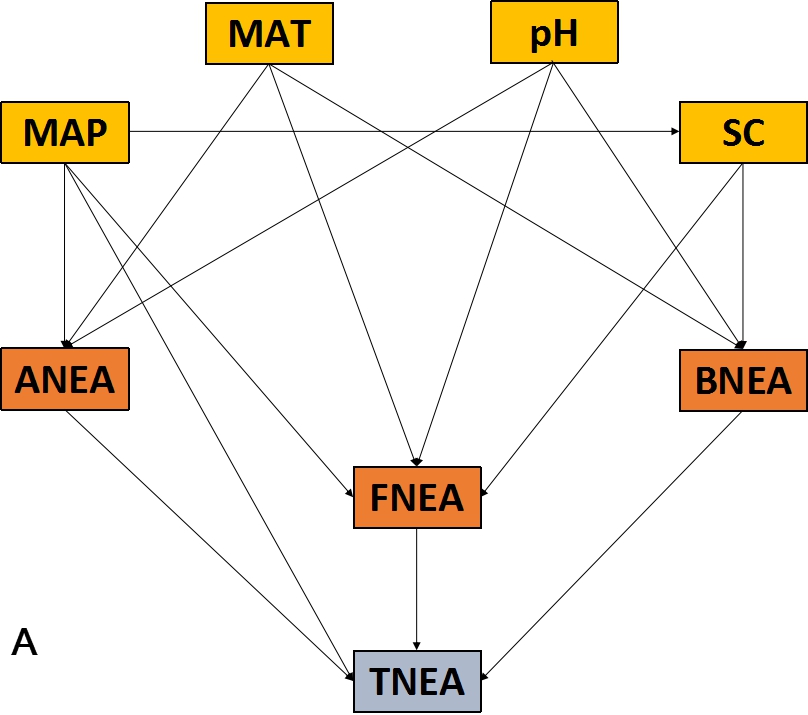


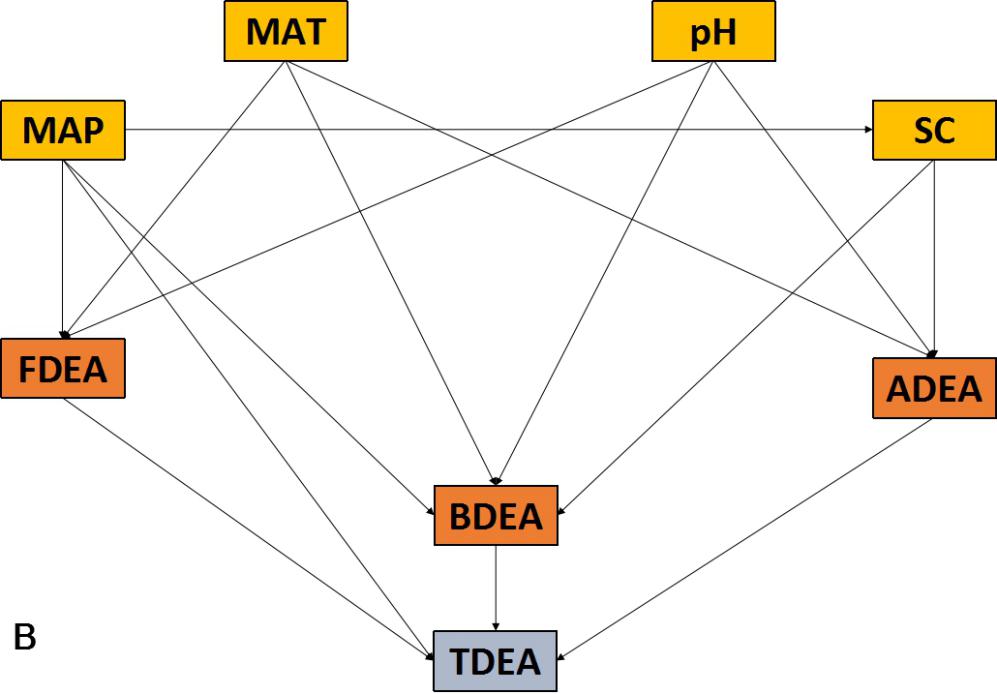


**Reference**

1. Rotthauwe J-H, Witzel K-P, Liesack W (1997) The ammonia monooxygenase structural gene amoA as a functional marker: molecular fine-scale analysis of natural ammonia-oxidizing populations. *Applied and Environmental Microbiology*, **63**, 4704-4712.
2. Francis CA, Roberts KJ, Beman JM, Santoro AE, Oakley BB (2005) Ubiquity and diversity of ammonia-oxidizing archaea in water columns and sediments of the ocean. *Proceedings of the National Academy of Sciences of the United States of America,* **102**, 14683-14688.
3. López-Gutiérrez JC, Henry S, Hallet S, Martin-Laurent F, Catroux G, Philippot L (2004) Quantification of a novel group of nitrate-reducing bacteria in the environment by real-time PCR. *Journal of microbiological methods,* **57**, 399-407.
4. Henry S, Baudoin E, López-Gutiérrez JC, Martin-Laurent F, Brauman A, Philippot L (2004) Quantification of denitrifying bacteria in soils by nirK gene targeted real-time PCR. *Journal of microbiological methods,* **59**, 327-335.
5. Throbäck IN, Enwall K, Jarvis Å, Hallin S (2004) Reassessing PCR primers targeting nirS, nirK and nosZ genes for community surveys of denitrifying bacteria with DGGE. *FEMS Microbiology Ecology*, **49**, 401-417.
6. Jones C M , Graf D R , Bru D , et al (2013). The unaccounted yet abundant nitrous oxide-reducing microbial community: a potential nitrous oxide sink[J]. *Isme Journal*, **7(2)**:417-426.
7. Long A, Song B, Fridey K, et al. (2015) Detection and diversity of copper containing nitrite reductase genes (nirK) in prokaryotic and fungal communities of agricultural soils[J]. FEMS microbiology ecology, **91(2)**.
8. Wei W, Isobe K, Shiratori Y, et al. (2015) Development of PCR primers targeting fungal nirK to study fungal denitrification in the environment[J]. Soil Biology and Biochemistry, **81**: 282-286.
9. Chen H, Yu F, Shi W. (2016) Detection of N2O-producing fungi in environment using nitrite reductase gene (nirK)-targeting primers[J]. Fungal biology, **120(12)**: 1479-1492.
